# Functionally-informed fine-mapping identifies genetic variants linking increased CHD1L expression and HIV restriction in monocytes

**DOI:** 10.1038/s41598-024-84817-y

**Published:** 2025-01-17

**Authors:** Riley H. Tough, Paul J. McLaren, The International Collaboration for the Genomics of HIV, Paul J. McLaren

**Affiliations:** 1 Sexually Transmitted and Bloodborne Infections Surveillance and Molecular Epidemiology, Sexually Transmitted and Bloodborne Infections Division at the JC Wilt Infectious Diseases Research Centre, National Microbiology Laboratories, Public Health Agency of Canada, Winnipeg, MB R3E 3L5 Canada; 2 Department of Medical Microbiology and Infectious Diseases, University of Manitoba, Winnipeg, MB R3E 0J9 Canada

**Keywords:** Genetic association study, HIV infections, Statistical methods

## Abstract

Human Immunodeficiency Virus Type 1 (HIV) set-point viral load is a strong predictor of disease progression and transmission risk. A recent genome-wide association study in individuals of African ancestries identified a region on chromosome 1 significantly associated with decreased HIV set-point viral load. Knockout of the closest gene, *CHD1L*, enhanced HIV replication in vitro in myeloid cells. However, it remains unclear if HIV spVL associated variants are associated with *CHD1L* gene expression changes. Here we apply a heuristic fine-mapping approach to prioritize combinations of variants that explain the majority of set-point viral load variance and identify variants likely driving the association. We assess the combined impact of these variants on *CHD1L* regulation using publicly available sequencing studies, and test the relationship between *CHD1L* expression and set-point viral load using imputed *CHD1L* expression from monocytes. Taken together, this work characterizes genetically regulated *CHD1L* expression and further expands our knowledge of *CHD1L*-mediated HIV restriction in monocytes.

**Supplementary Information:**

The online version contains supplementary material available at 10.1038/s41598-024-84817-y.

## Introduction

Human Immunodeficiency Virus Type 1 remains a major risk to global health with approximately 1.3 million new infections in 2022^1^. The amount of HIV RNA circulating in the blood during chronic phase of infection, referred to as HIV set-point viral load (spVL), is a strong predictor of transmission risk^2,3^ with each 1 log_10_ increase in HIV RNA increasing transmission risk by up to 2.9-fold^4,5^. However, when an individual achieves viral suppression through antiretroviral therapy (ART), they are unable to cause infection through sexual transmission^6–9^. Therefore, in the absence of a vaccine or functional cure, limiting HIV viral load in people living with HIV is critical to reducing new infections.

Antiretroviral therapy has greatly improved the quality of life of people living with HIV and limited the spread of the virus^9^, but the accumulation of escape mutations may threaten the long-term use of ART^10^. Antivirals targeting host proteins essential for the viral life cycle have shown a greater barrier to the development of resistance compared to direct-acting antivirals and may assist in maintaining the sustained efficacy of HIV treatments^11,12^. Therefore, an effective strategy at ensuring the long-term efficacy of ART could include the addition of safe, effective therapeutics that reduce the likelihood of HIV antiviral resistance.

Large-scale host knockout studies have identified hundreds of HIV host dependency and restrictions factors^13–17^. These studies have provided valuable insight to how host genes can modulate HIV disease in vitro and identified robust candidates for therapeutic intervention. Genetic association studies have also provided insight to how host genetic variation is associated with HIV disease progression at the population level^18,19^. However, many host genomic studies do not accurately represent global genetic diversity, focusing mainly on individuals of European ancestry^20^. In the context of HIV disease, where the burden of illness is predominately in African populations and approximately 25% of variance of HIV spVL is attributable to host genetics^21^, there is an opportunity to identify new HIV host dependency and restriction factors in underrepresented populations.

A recent genome-wide association study (GWAS) of HIV spVL with individuals of African ancestries identified a novel region on chromosome 1 that is significantly associated with decreased HIV spVL^22^. The top SNP from this study, rs59784663-A-G, is present in African populations ranging from 4 to 12%, and less than 0.1% in European and Asian populations^23,24^. Variants in high linkage disequilibrium (LD) in the region overlap three coding genes *CHD1L*,* PRKAB2*, and *FMO5*, but this region had not been previously implicated in HIV host genomic studies with European populations^25^. Initial investigations focused on the closest gene, *CHD1L*, a helicase involved in DNA repair for its potential role in HIV integration^26^. In vitro knockout studies in myeloid cells, but not Jurkat T cells, showed increasing HIV p24 production in cells depleted of *CHD1L* and in the U2OS model system showed *CHD1L* dose-dependent HIV restriction^22^. However, the genetic mechanisms underlying this effect remains unknown.

Here we perform a heuristic functionally informed fine-mapping approach in the African ancestry sample of the International Collaboration for the Genomics of HIV (ICGH) cohort to identify which variants likely drive *CHD1L* gene expression changes and the overall impact of genetic variation on *CHD1L* expression. Using chromatin states, regulatory motifs, and gene expression profiles, we tested the association of these variants in blood tissues from individuals of African ancestry. Identification of these functional signals provides insight to genetically regulated *CHD1L* expression across multiple immune cell populations, suggesting that the chromosome 1 signal is associated with increased *CHD1L* expression in monocytes.

## Results

### Fine-mapping of HIV spVL locus implicates multiple causal variants

Genome-wide association studies identify genomic regions associated with a particular phenotype, but do not inherently provide information on causal variants or functional consequences. Therefore, we sought to prioritize candidate variants driving the HIV spVL association in chromosome 1 by identifying overlap between regulatory variants and variants that explain the majority of HIV spVL variance. To do this, we accessed genotype data from the African ancestry component of the ICGH spVL cohort (*N* = 3,886) and selected genetic variants in LD to the top associated HIV spVL variant in the chromosome 1 region, rs59784663-A-G (r^2^ ≥ 0.2, *N* = 121) and annotated them with HaploRegv4.2^27^. Genetic variants were characterized as functionally relevant if they overlapped known or imputed chromatin states, H3K4me1, H3K4Me3, H3K27ac, H3K9ac, DNase peaks, and/or bound protein regions in 24 blood tissues including primary mononuclear, T regulatory, T helper, T CD8 + naive, monocytes, hematopoietic stem, natural killer, neutrophils, and/or K562 cell line (full tissue list available in Supplementary Table S1). Known expression quantitative trait loci (eQTL) for *CHD1L* in any tissue from the Genotype-Tissue Expression (GTEx)^28^ database were also included. This analysis identified 84 functional HIV spVL associated variants within the chromosome 1 region that overlap within known or predicted regulatory regions (Supplementary Table S2).

To identify combinations of likely causal variants, we applied the Bayesian Imputation Based Association Mapping (BIMBAM) Bayesian framework to the functional variants and calculated the Bayes Factors (BF) for multi-SNP models to be driving the association^29^. We observed significant explanatory potential when considering 1-SNP (log_10_BF = 6.52), 2-SNP (log_10_BF = 6.74), and 3-SNP (log_10_BF = 6.75) models, with increasing significance of multi-SNP models indicating that a combination of two or three genetic variants are most likely responsible for driving the association. In the 2-SNP model, the most significant combination of variants was rs7525622-G-A with rs73004025-C-T (log_10_BF = 8.73) followed by rs72999655-A-G with rs7525622-G-A (log_10_BF = 8.583). In the 3-SNP model, the most significant combination of variants was rs72999655-A-G, rs7525622-G-A, and rs73004025-C-T (log_10_BF = 8.663). Therefore, we moved forward with the 3-SNP model to increase the likelihood of identifying a causal signal (Supplementary Table S3). The minor allele of these variants were significantly associated with reduced HIV spVL and had varying degrees of linkage to the top associated single SNP, rs59784663-A-G (Table 1). Next, we performed a linear regression analysis comparing the variance explained by the multi-SNP model, rs7525622-G-A, rs73004025-C-T, and rs72999655-A-G dosage against rs59784663-A-G, controlling for sex and principle component (PC) 1. This analysis showed that the combination of the three variants explained significantly more variance of HIV spVL than rs59784663-A-G alone (df = 6, Sum of Sq = 13.551, *p* ≤ 0.027).

**Table 1:** Genetic variants in the chromosome 1 region with the strongest association to HIV set-point viral load in a multi-SNP model using BIMBAM.

Variant	HIV spVL Beta^A, B^	HIV spVL *P*-Value^B^	*r*^2^ to rs59784663
rs72999655-A-G	− 0.30	9.09 × 10^− 10^	0.99
rs7525622-G-A	− 0.21	3.98 × 10^− 9^	0.46
rs73004025-C-T	− 0.29	6.36 × 10^− 9^	0.90

^A^Direction corresponds to the effect of the minor allele. ^B^Values reflect the association between genetic variants and HIV set-point viral load in African populations from McLaren et al.^22^.

**Fig. 1:**
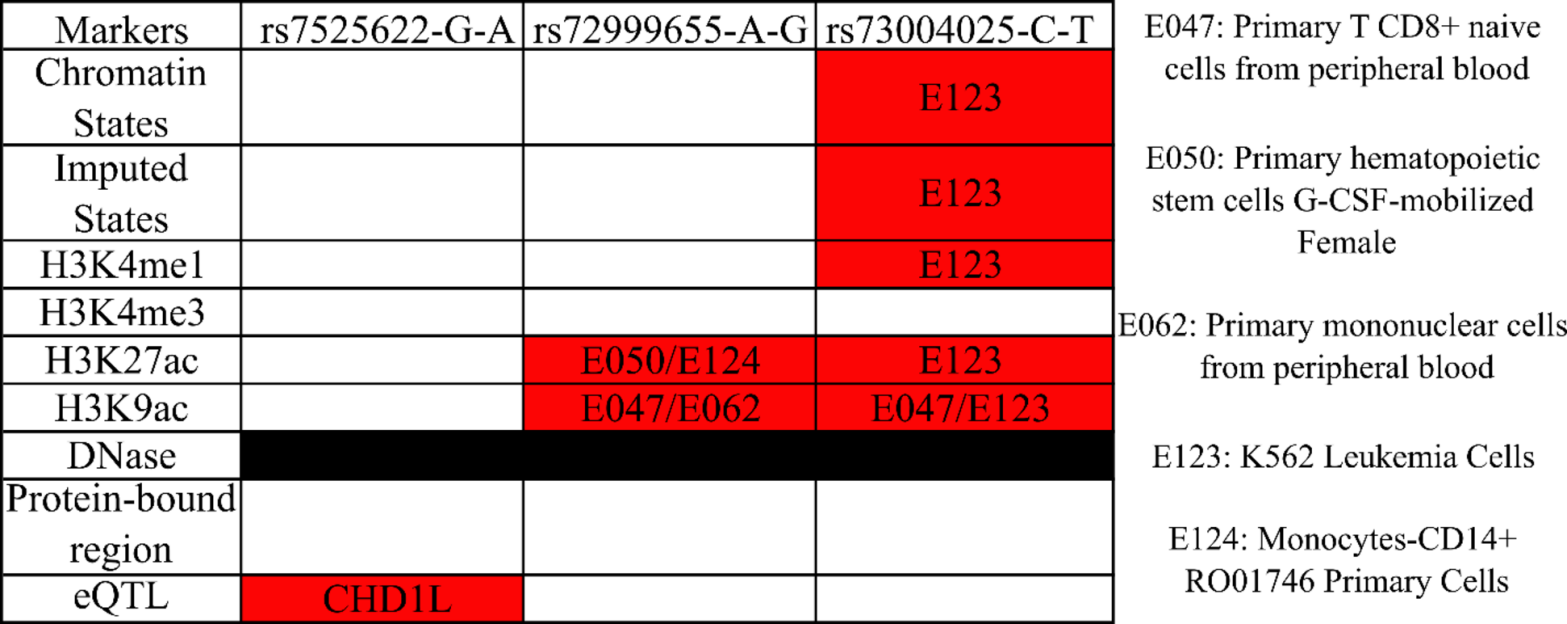
Overlap of putative causal variants and their corresponding functional annotations from HaploRegv4.2.

Two of the variants identified as likely causal, rs73004025-C-T and rs72999655-A-G, overlap with known chromatin states and histone modifications in blood tissues from the Encyclopedia of DNA Elements (ENCODE) and Roadmap Epigenetics databases (Fig. 1). The variant rs73004025 is located downstream of *CHD1L* and was associated with altered methylation states in K562 and CD8 + T cells^30^. The variant rs72999655-A-G overlaps with *CHD1L* transcripts (ENST00000369258.8, ESNT00000361293.10, and ENST00000369259.4) and was associated with altered methylation profiles in primary hematopoietic stem cells, monocytes, primary CD8 + T cells, and primary mononuclear cells from peripheral blood. The variant rs7525622-C-T is located downstream of *CHD1L* and was identified to be an eQTL associated with increased *CHD1L* expression in cultured fibroblasts (*p* ≤ 4.4 × 10^− 11^, NES = 0.24) and skeletal muscle (*p* ≤ 1.4 × 10^− 10^, NES = 0.43)^31^.

**Fig. 2:**
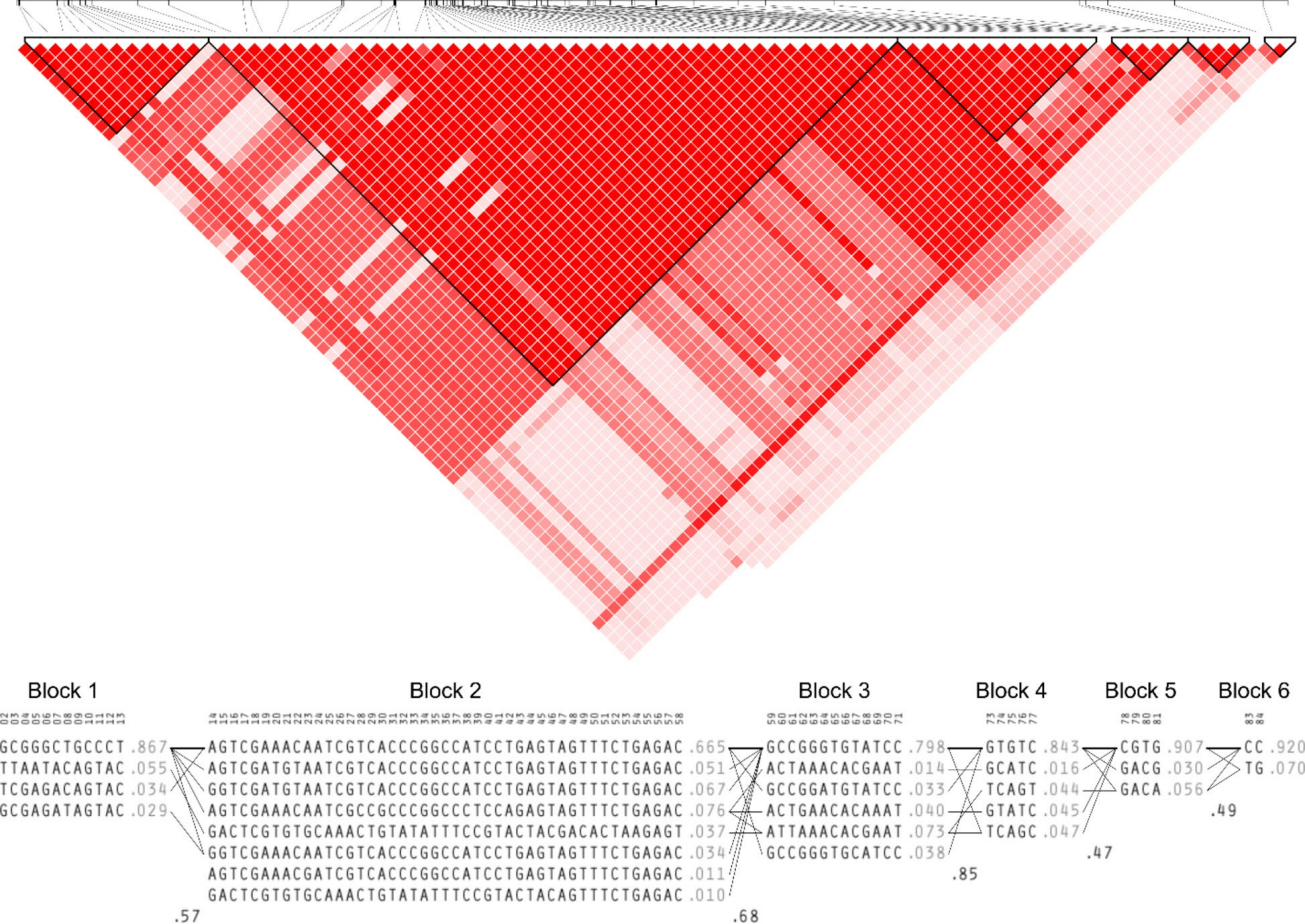
Linkage disequilibrium patterns of functional variants in the chromosome 1 region. The heat map depicts LD (D’) between functional chromosome 1 variants, with darker shades representing stronger LD between variants. Haplotypes are represented if they were present in at least 1% of samples.

We then sought to characterize the LD relationship between these variants; we clustered the 84 functional variants into haplotypes using Haploview 1.4.4 which identified six unique haplotypes and 3 non-linked variants (Fig. 2, Supplementary Table S4). Notably, the variants identified through BIMBAM (rs72999655-A-G, rs7525622-G-A, and rs73004025-C-T), were the most significant variants associated within blocks 2, 3, and 4, respectively. Taken together, these functional annotations suggest that these variants may have the potential to impact *CHD1L* expression in blood tissues but the overall magnitude and direction of effect is unclear.

### Increased CHD1L expression is significantly correlated with decreased HIV spVL

**Fig. 3:**
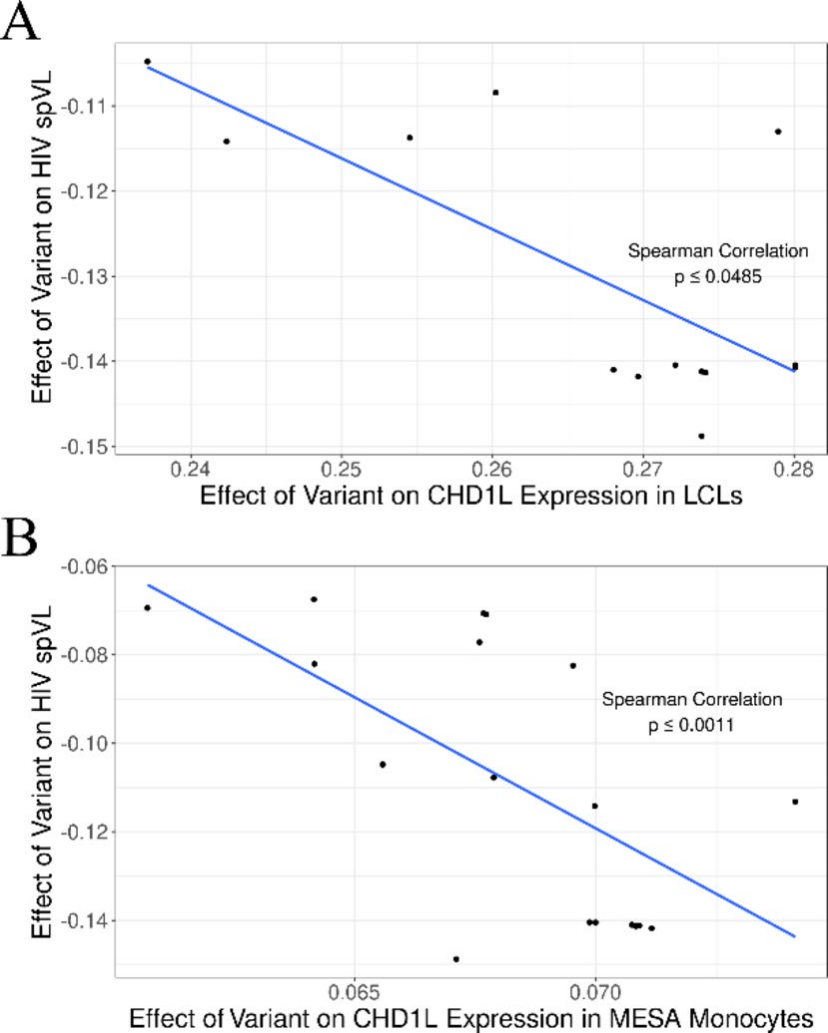
Correlations of *CHD1L* eQTLs and their effect on HIV spVL. Linear regression analyses were performed on functional variants that are in haplotypes with candidate causal GWAS variants rs72999655-A-G, rs7525622-G-A, and rs7300425-C-T. (**A**) Analysis of lymphoblastoid cell line *CHD1L* eQTLs from the African Functional Genomics Resource cohort^52^. (**B**) Analysis of monocyte *CHD1L* eQTLs from the Multi-Ethnic Study of Atherosclerosis cohort^53^. The blue line depicts the linear regression model of HIV spVL β and *CHD1L* eQTL β.

In order to determine the impact of genetic variants on *CHD1L* expression, we investigated large African eQTL studies to determine potential functions of our candidate variants. We accessed lymphoblastoid cell line (LCL) eQTL summary statistics from the African Functional Genomics Resource (AFGR) and tested whether the fine-mapped variants are associated with *CHD1L* gene expression changes. We identified 14 overlapping variants that have a significant correlation between increased *CHD1L* expression and decreased HIV spVL (*p* ≤ 0.049) (Fig. 3A). We next accessed data on monocyte eQTLs from the Multi-Ethnic Study of Atherosclerosis (MESA) cohort and observed a significant correlation between eQTLs increasing *CHD1L* expression and decreased HIV spVL (*p* ≤ 0.0022) (Fig. 3B). Taken together, these results suggest the chromosome 1 signal is associated with increased *CHD1L* expression and support prior in vitro results that suggested increasing *CHD1L* decreases HIV replication.

### Functional GWAS variants are associated with altered CHD1L transcription start site methylation

**Fig. 4:**
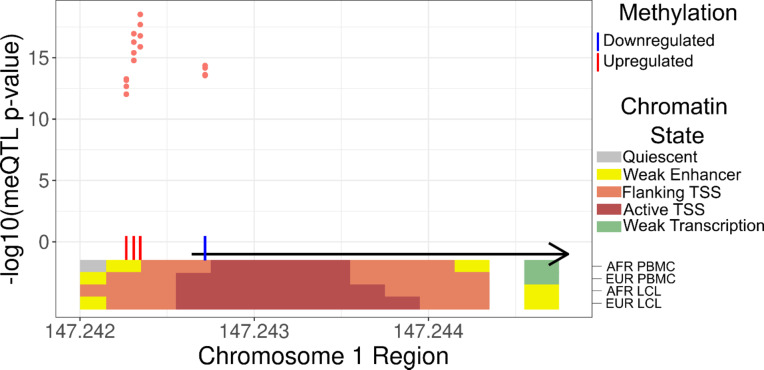
Methylation quantitative trait loci of HIV spVL associated SNPs in African populations. Each data point (pink) represents a meQTL in the chromosome 1 region in linkage disequilibrium (r^2^ = 1) with rsrs72999655-A-G. The vertical bars show whether the meQTL is associated with hypermethylation (red) or hypomethylation (blue). The black arrow represents the *CHD1L* transcript from RefSeq hg38. Overlapping colored bars represent chromatin states obtained from ENCODE (African PBMC: ENCFF863IVL; European PBMC: ENCFF235OWS; African LCLs: ENCFF747OPJ; European LCLs: ENCFF070KWN).

Next, we sought to determine whether HIV spVL associated variants in the chromosome 1 region may affect *CHD1L* expression through altered methylation profiles using peripheral blood leukocyte methylation quantitative trait loci (meQTL) from the Genetic Epidemiology Network of Arteriopathy (GENOA) cohort. There were twelve variants, rs72999634-A-G, rs72999637-T-C, rs72999638-T-A, rs72999639-T-C, rs72999640-C-T, rs72999646-T-A, rs72999648-G-A, rs72999655-A-G, rs72999656-C-T, rs7526114-G-A, rs59213667-G-A, and rs7535451-A-G, which tagged hypermethylation at four CpG sites (cg07572770, cg11120551, cg15022772, cg21917976) and hypomethylation at a single CpG site (cg25205988) (Fig. 4, Supplementary Table S5). The minor alleles of each variant were associated with methylation changes and were associated with decreased HIV spVL. These variants were in perfect LD (r^2^ = 1.0) with rs72999655-A-G, and since there variants are inherited together, we assessed their functional as a single effect.

To assess the impact of these CpG sites on *CHD1L*, we obtained African and European chromatin states from the ENCODE database for peripheral blood mononuclear cells (PBMCs) and LCLs. The hypomethylated CpG site, cg25205988, corresponds to an active transcription start site (TSS) in 29 blood cell types, an active enhancer in two cell types, flanking TSS in 11 cell types, and upstream flanking transcription start site in neutrophils^32^. The enrichment of DNA methylation within the *CHD1L* promoter suggests that these HIV spVL associated variants in African populations likely regulate *CHD1L* expression.

### Variants in the chromosome 1 region are associated with increased CHD1L expression

Previously, we tested the association of individual *CHD1L* eQTLs from the AFGR cohort against the effect of HIV spVL from the ICGH HIV spVL cohort. While this is informative on the effects of individual variants, it does not provide information on the relationship of *CHD1L* and HIV spVL in an individual sample. To test the association between HIV spVL and *CHD1L* expression in the same sample, genetically regulated *CHD1L* gene expression was imputed on individual-level genotypes from the ICGH cohort using PrediXcan imputation models in monocytes from African Americans in the MESA cohort.

**Fig. 5:**
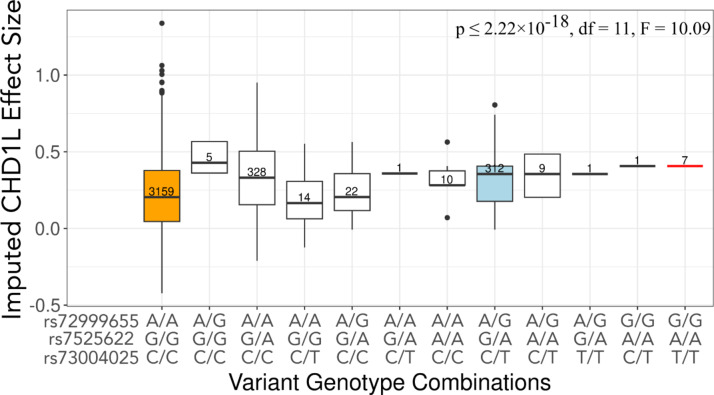
Association between rs72999655-A-G, rs7525622-G-A, and rs73004025-C-T genotype with PrediXcan imputed *CHD1L* expression. The orange box represents homozygous reference at all alleles, light blue as heterozygous at all alleles, and red is homozygous alternate at all alleles. Significance was determined using linear regression controlling for PC1 and sex.

**Fig. 6:**
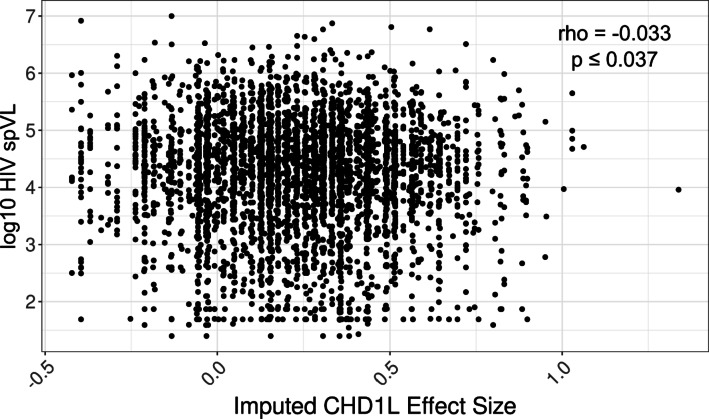
Spearman correlation of imputed *CHD1L* genetic variant weights against HIV set-point viral load in 3,886 individuals of African ancestry as part of the International Collaboration of Genomics for HIV. CHD1L weights were imputed using monocyte eQTLs from the Multi-Ethnic Study of Atherosclerosis cohort and PrediXcan on individual-level genotype data.

When we compared the dosage of likely causal variants to imputed *CHD1L* expression, controlling for sex and PC1, we saw that protective HIV spVL associated variant alleles were significantly correlated with increased *CHD1L* expression (*p* ≤ 2.22 × 10^− 18^, df = 11, F = 10.09) (Fig. 5). We next tested the relationship between *CHD1L* and HIV spVL, observing that increased *CHD1L* is significantly associated with decreased HIV spVL (*p* ≤ 0.04, df = 1, F = 4.1867) (Fig. 6). Within this data, we see that individuals with the putative causal variants in the chromosome 1 region have higher predicted levels of *CHD1L* expression in monocytes. These results suggest there is a strong likelihood that the variants rs72999655-A-G, rs73004025-C-T, and rs7525622-G-A, and/or their associated haplotypes, are driving the association between HIV spVL and increased *CHD1L* gene expression.

## Discussion

HIV infection remains a life-long illness that requires people living with HIV to take medication to control replication of the virus and reduce the risk of transmission. Following a large African GWAS, it was discovered that increased *CHD1L* expression inhibits HIV replication in individuals of African ancestry in a dose-dependent manner^22^. The study applied a penalized regression fine-mapping model using the Probabilistic Annotation INTegratOR (PAINTOR) framework to variants in the chromosome 1 region and identified 16 variants as part of a 95% credible set of candidate causal variants for regulation of HIV spVL. However, it is unclear whether HIV spVL associated SNPs in the chromosome 1 region are associated with *CHD1L* gene expression and, if so, the direction of the effect. In this study, we employ a heuristic and Bayesian fine-mapping approach that identifies three genetic variants: rs72999655-A-G, rs7525622-G-A, and rs73004025-C-T, which explain significantly more HIV spVL variance than the lead GWAS SNP, rs59784663-A-G.

The majority of participants in the HIV spVL GWAS were of African American ancestry (*N* = 2868) and genetically represent an admixed African-European population. This unique genetic architecture results in reduced LD and increased haplotype diversity within the population which can make identifying a causal signal challenging^33–35^. Therefore, we employed a heuristic approach by prioritizing variants in LD to the top associated variant, rs59784663-A-G, and then applying a Bayesian and penalized regression model to determine the likely number of causal variants and identify variants that explain the majority of HIV spVL variance. We identify three genetic variants: rs72999655-A-G, rs7525622-G-A, and rs73004025-C-T, which explain significantly more variance of HIV spVL than rs59784663-A-G. The putatively causal variant rs7525622-G-A is a *CHD1L* eQTL in fibroblasts (NES = 0.24, *p* < 4.4 × 10^− 11^) and skeletal muscle (NES = 0.43, *p* < 1.4 × 10^− 10^) from the GTEx database^36^, however the variant was not a statistically significant eQTL in the MESA or AGFR cohorts. This is likely due to the differences in tissue types and/or samples sizes between the GTEx, MESA and AGFR data sets. The GTEx database contains 802 white and 122 African American donors, the MESA cohort had monocytes from 352 European and 233 African individuals, and the AFGR cohort had LCLs from 599 individuals of African ancestry. While not statistically significant, rs7525622-G-A had the same direction of effect as GTEx tissues in the MESA (β = 0.056, FDR ≥ 0.19) and AFGR cohort (β = 0.19, *p* ≤ 0.0042); suggesting that larger sample sizes are necessary for concluding an effect in these cell types.

While rs73004025-C-T overlaps with altered H3K9ac in primary CD8 + T cells and chromatin states in K562 cells, as determined by haploR^37^ in ENCODE data^27,38^; this variant is not an eQTL in GTEx, AGFR, or MESA and is not an meQTL in ENCODE. The variant rs72999655-A-G is in perfect LD (r^2^ ≥ 1) with 11 other variants tested here, all of which are associated with decreased HIV spVL, and are significantly associated with hypermethylation of three CpG sites flanking the *CHD1L* active TSS and associated with hypomethylation of the *CHD1L* TSS. Hypermethylated promoter regions have traditionally been associated with transcriptional repression while hypomethylated regions are associated with increased transcriptional activity^39,40^. However, recent studies have suggested that hypermethylation may cause increased expression of nearby genes in some instances^41,42^. Given the strong association between variants and altered methylation states, and that methylation states are cell-type specific, we hypothesize that the chromosome 1 region may regulate *CHD1L* in a cell-type specific manner.

Here we identify a subset of genetic variants in the chromosome 1 region that explain more variance than the lead GWAS variant, rs59784663-A-G, and that the presence of these variants were associated with increased *CHD1L* expression in monocytes. However, we acknowledge that the monocyte training dataset from the MESA cohort uses gene expression in healthy individuals but that expression profiles in monocytes are significantly altered during HIV infection^43^. Therefore, we highlight the need for large-scale sequencing studies in people living with HIV and individuals of African ancestry to assess the impact of genetically regulated *CHD1L* expression. This study provides a baseline for understanding the genetically regulated impact of *CHD1L*-mediated HIV inhibition, but further investigations into *CHD1L* expression in HIV-relevant cell types, induction of likely causal variants to confirm regulatory potential, and determining the mechanism of action of *CHD1L* will be necessary to assess the potential of *CHD1L* as a novel HIV therapeutic target.

## Methods

### GWAS cohort, genotyping, and quality control

Genomic data was obtained from ten independent GWAS and permission for use was obtained from the ICGH cohort^22,25,44^. Detailed methods, including a description of the population cohort, sequencing, quality control, imputation and association analyses for the HIV set-point viral load GWAS were performed in accordance with the relevant guidelines and regulations, as described previously^22^. For this meta-analysis, PCs were calculated for the entire cohort using EIGENSTRAT within EIGENSOFT (v.7.2.1) to control for population stratification^45^. Significant PCs were determined using a linear regression analysis against HIV spVL at *p* < 0.05.

### Functional annotation of GWAS variants

Linkage disequilibrium patterns of variants in the chromosome 1 region were determined using plink (v1.9)^46^ and individual-level genotype data from the ICGH cohort^22^. HIV spVL associated SNPs in LD to rs59784663-A-G (r^2^ ≥ 0.2) were considered for follow-up analyses. Variant annotation was performed using HaploReg v4.2 to integrate experimental annotations from over 127 epigenomes in the ENCODE and Roadmap Epigenetics projects designed to aid in fine-mapping likely causal GWAS variants and determining functional impact of genetic variants^27,30,47^. We queried HaploRegv4.2 using haploR version 1.4.4 to directly annotate genetic variants with overlapping cell-type specific chromatin states, H3K4me1, H3K4Me3, H3K27ac, H3K9ac, DNase, eQTLs, and protein binding sites in 24 blood tissues to better account for elements associated with HIV infection (complete list of tissues in Supplementary Table S1)^27,37^. Genetic variants that had at least one functional marker in blood tissues were selected for follow-up.

### Statistical prioritization of genetic variants

Variants potentially driving the genetic association with HIV spVL were filtered for at least one functional annotation from the HaploReg analysis and used as input to the BIMBAM Bayesian modeling framework^29,48^. BIMBAM computes multi-SNP Bayes factors of genetic variants against a phenotype of interest, evaluating up to N possible models. Variant dosages were calculated as the mean genotype, as recommended by developers^48^. The number of putative causal variants was determined using the BIMBAM model, where adding more causal variants did not improve the model’s performance. Significance of variant combinations was defined as log_10_ BF ≥ 3.5 as defined by Servins and Stephens, 2007^29^.

### Identification of likely causal variants

Variants in the chromosome 1 region exhibited high inter-SNP LD patterns, so we clustered variants into haplotype blocks using Haploview v4.2 to prevent oversampling variants in high LD^49,50^. Haplotypes were created using the model as described by Gabriel et al.^51^, for variants with a minor allele frequency greater than 0.04. Haplotypes (*N* = 3) that had at least one GWAS significant variant were selected for statistical modelling. We generated a linear regression model of rs59784663-A-G dosage and performed a chi-square test against a linear regression model of rs72999655-A-G, rs7525622-G-A, and rs73004025-C-T in the ICGH combined cohort, controlling for sex and PC1 in both models. Statistical analyses and graphing was performed using R (version 4.1.2). Significance was determined against *p* ≤ 0.05.

### Identification of CHD1L eQTLs from publicly available summary statistics

We obtained LCL cell line eQTL summary statistics originating from 599 individuals of African ancestry from 1000 Genomes Populations (GWD *N* = 112; ESN *N* = 97; MSL *N* = 83; YRI *N* = 41; LWK *N* = 96; MKK *N* = 164) as part of the AFGR cohort^52^. We filtered significant eQTLs (FDR < 0.05) for those that overlapped the functional HIV spVL associated SNPs and limited eQTLs to those influencing *CHD1L* expression. Association between *CHD1L* eQTLs and HIV spVL β was assessed using a linear regression and two-sided spearman approximate correlation in R (version 4.1.2).

Additionally, summary statistics for eQTLs in monocytes were obtained from the MESA cohort for 233 African Americans, 352 Hispanic, and 578 individuals of European ancestry^53^. Significant eQTLs (FDR < 0.05) from the combined population meta-analysis were filtered for functional HIV spVL associated SNPs. Association between *CHD1L* eQTLs and HIV spVL β was tested using a linear regression model and two-sided spearman approximate correlation.

### Identification of CHD1L meQTLs from publicly available summary statistics

Summary statistics for meQTLs in peripheral blood leukocytes were obtained from the GENOA cohort for 961 individuals of African ancestry^54^. Variants that were significant meQTLs (FDR < 0.05) were filtered for overlap with functional HIV spVL associated SNPs. Chromatin states of PBMCs from individuals of African (ENCFF863IVL) and European ancestry (ENCFF235OWS), as well as LCLs from individuals of African (ENCFF747OPJ) and European ancestry (ENCFF070KWN), were obtained from ENCODE^55^. Hypermethylated sites were defined as β ≥ 0.7 and hypomethylated as β ≤ 0.3^56^. Visualization of the chromatin state, *CHD1L* transcript (RefSeq hg38), and methylation sites were performed using R (v4.1.2).

### Transcriptome-wide association study for HIV spVL

In order to test the association between *CHD1L* expression and HIV spVL in the same sample, we imputed *CHD1L* expression using PrediXcan^57^ on individual-level genotypes from the African HIV spVL GWAS (*N* = 3,886) and African American monocyte eQTL data from the MESA cohort. Training data was limited to individuals of African Ancestry to reduce effects of population stratification^53^. Imputed expression of *CHD1L* was compared for individuals with allelic combinations of rs59784663A-G, rs72999655-A-G, rs7525622-G-A, and rs73004025-C-T. Statistical significance was determined between allelic dosages using a Wilcoxon rank-sum test.

## Electronic supplementary material

Below is the link to the electronic supplementary material.

Supplementary Material 1

Supplementary Material 2

## Data Availability

Access to individual-level genotyping data is restricted to investigators from institutions that join the ICGH cohort by signing the ICGH collaboration agreement, which is obtainable on request (jacques.fellay@epfl.ch). Owing to the highly sensitive nature of the HIV diagnostic of all study participants, the risk associated with potential re-identification was deemed to be very high by the Institutional Review Boards, preventing broader sharing of individual-level data. Summary statistics of meQTLs in peripheral blood leukocytes is publicly available from https://github.com/smontgomlab/AFGR. Summary statistics of monocyte eQTL models and PrediXcan models is publicly available from https://github.com/WheelerLab/DivPop. Additional datasets used and/or analysed within this manuscript are available from the corresponding author on reasonable request (paul.mclaren@phac-aspc.gc.ca).

## References

[1] The path that ends AIDS: UNAIDS Global AIDS Update 2023. *Geneva Jt. United Nations Program. HIV/AIDS* (2023).

[2] Quinn, T. C. et al. Viral load and heterosexual transmission of human immunodeficiency virus type 1. Rakai Project Study Group. *N Engl. J. Med.* **342**, 921–929 (2000). 10738050 10.1056/NEJM200003303421303

[3] Fideli, U. S. et al. Virologic and immunologic determinants of heterosexual transmission of human immunodeficiency virus type 1 in Africa. *AIDS Res. Hum. Retroviruses*. **17**, 901–910 (2001). 11461676 10.1089/088922201750290023 PMC2748905

[4] Modjarrad, K., Chamot, E. & Vermund, S. H. Impact of small reductions in plasma HIV RNA levels on the risk of heterosexual transmission and disease progression. *AIDS* **22**, 2179–2185 (2008). 18832881 10.1097/QAD.0b013e328312c756 PMC2661869

[5] Hughes, J. P. et al. Determinants of per-coital-act HIV-1 infectivity among African HIV-1-serodiscordant couples. *J. Infect. Dis.* **205**, 358–365 (2012). 22241800 10.1093/infdis/jir747 PMC3256946

[6] Cohen, M. S. et al. Prevention of HIV-1 infection with early antiretroviral therapy. *N Engl. J. Med.* **365**, 493–505 (2011). 21767103 10.1056/NEJMoa1105243 PMC3200068

[7] Rodger, A. J. et al. Sexual activity without condoms and risk of HIV transmission in serodifferent couples when the HIV-positive partner is using suppressive antiretroviral therapy. *JAMA* **316**, 171–181 (2016). 27404185 10.1001/jama.2016.5148

[8] LeMessurier, J. et al. Risk of sexual transmission of human immunodeficiency virus with antiretroviral therapy, suppressed viral load and condom use: a systematic review. *CMAJ* **190**, E1350–E1360 (2018). 30455270 10.1503/cmaj.180311 PMC6239917

[9] Rodger, A. J. et al. Risk of HIV transmission through condomless sex in serodifferent gay couples with the HIV-positive partner taking suppressive antiretroviral therapy (PARTNER): final results of a multicentre, prospective, observational study. *Lancet (London England)*. **393**, 2428–2438 (2019). 31056293 10.1016/S0140-6736(19)30418-0 PMC6584382

[10] HIV drug resistance report 2019. *Geneva World Heal. Organ.* (2019).

[11] Delang, L., Vliegen, I., Froeyen, M. & Neyts, J. Comparative study of the genetic barriers and pathways towards resistance of selective inhibitors of hepatitis C virus replication. *Antimicrob. Agents Chemother.* **55**, 4103–4113 (2011). 21709100 10.1128/AAC.00294-11 PMC3165355

[12] Roa-Linares, V. C., Escudero-Flórez, M., Vicente-Manzanares, M. & Gallego-Gómez, J. C. Host cell targets for unconventional antivirals against RNA viruses. *Viruses* **15**, (2023). 10.3390/v15030776 PMC10058429 36992484

[13] Zhou, H. et al. Genome-scale RNAi screen for host factors required for HIV replication. *Cell. Host Microbe*. **4**, 495–504 (2008). 18976975 10.1016/j.chom.2008.10.004

[14] Zhu, J. et al. Comprehensive identification of host modulators of HIV-1 replication using multiple orthologous RNAi reagents. *Cell. Rep.* **9**, 752–766 (2014). 25373910 10.1016/j.celrep.2014.09.031 PMC4926641

[15] Schott, K. & König, R. Picking the Survivor! CRISPR reveals HIV dependency factors. *Trends Microbiol.* **25**, 243–245 (2017). 28233621 10.1016/j.tim.2017.02.004

[16] Park, R. J. et al. A genome-wide CRISPR screen identifies a restricted set of HIV host dependency factors. *Nat. Genet.* **49**, 193–203 (2017). 27992415 10.1038/ng.3741 PMC5511375

[17] Ohainle, M. et al. A virus-packageable CRISPR screen identifies host factors mediating interferon inhibition of HIV. *Elife* **7**, 1–32 (2018). 10.7554/eLife.39823 PMC6286125 30520725

[18] Fellay, J., Shianna, K. V., Telenti, A. & Goldstein, D. B. Host genetics and HIV-1: the final phase? *PLoS Pathog*. **6**, 1–9 (2010). 10.1371/journal.ppat.1001033 PMC2954832 20976252

[19] Kenney, A. D. et al. Human genetic determinants of viral diseases. *Annu. Rev. Genet.* **51**, 241–263 (2017). 28853921 10.1146/annurev-genet-120116-023425 PMC6038703

[20] Popejoy, A. B. & Fullerton, S. M. Genomics is failing on diversity. *Nature* **538**, 161–164 (2016). 27734877 10.1038/538161a PMC5089703

[21] McLaren, P. J. et al. Polymorphisms of large effect explain the majority of the host genetic contribution to variation of HIV-1 virus load. *Proc. Natl. Acad. Sci. U S A*. **112**, 14658–14663 (2015). 26553974 10.1073/pnas.1514867112 PMC4664299

[22] McLaren, P. J. et al. Africa-specific human genetic variation near CHD1L associates with HIV-1 load. *Nature* **620**, 1025–1030 (2023). 37532928 10.1038/s41586-023-06370-4 PMC10848312

[23] Karczewski, K. J. et al. Variation across 141,456 human exomes and genomes reveals the spectrum of loss-of- function intolerance across human protein-coding genes. (2019). 10.1101/531210

[24] Karczewski, K. J. et al. The mutational constraint spectrum quantified from variation in 141,456 humans. *Nature* **581**, 434–443 (2020). 32461654 10.1038/s41586-020-2308-7 PMC7334197

[25] McLaren, P. J. et al. Association study of common genetic variants and HIV-1 acquisition in 6,300 infected cases and 7,200 controls. *PLoS Pathog*. **9**, e1003515 (2013). 23935489 10.1371/journal.ppat.1003515 PMC3723635

[26] Tsuda, M. et al. ALC1/CHD1L, a chromatin-remodeling enzyme, is required for efficient base excision repair. *PLoS One*. **12**, e0188320 (2017). 29149203 10.1371/journal.pone.0188320 PMC5693467

[27] Ward, L. D. & Kellis, M. HaploReg v4: systematic mining of putative causal variants, cell types, regulators and target genes for human complex traits and disease. *Nucleic Acids Res.* **44**, D877–D881 (2016). 26657631 10.1093/nar/gkv1340 PMC4702929

[28] Consortium, G. et al. Genetic effects on gene expression across human tissues. *Nature*. **550**, nature24277 (2017). 10.1038/nature24277 PMC5776756 29022597

[29] Servin, B. & Stephens, M. Imputation-based analysis of association studies: candidate regions and quantitative traits. *PLoS Genet.* **3**, e114 (2007). 17676998 10.1371/journal.pgen.0030114 PMC1934390

[30] Davis, C. A. et al. The encyclopedia of DNA elements (ENCODE): data portal update. *Nucleic Acids Res.* **46**, D794–D801 (2018). 29126249 10.1093/nar/gkx1081 PMC5753278

[31] Kim-Hellmuth, S. et al. Cell type–specific genetic regulation of gene expression across human tissues. *Science* **369**, (2020). 10.1126/science.aaz8528 PMC8051643 32913075

[32] Dong, S. & Boyle, A. P. Prioritization of regulatory variants with tissue-specific function in the non-coding regions of human genome. *Nucleic Acids Res.* **50**, e6 (2022). 34648033 10.1093/nar/gkab924 PMC8754628

[33] Zakharia, F. et al. Characterizing the admixed African ancestry of African americans. *Genome Biol.* **10**, R141 (2009). 20025784 10.1186/gb-2009-10-12-r141 PMC2812948

[34] Martin, E. R. et al. Properties of global- and local-ancestry adjustments in genetic association tests in admixed populations. *Genet. Epidemiol.* **42**, 214–229 (2018). 29288582 10.1002/gepi.22103 PMC5811405

[35] Sul, J. H., Martin, L. S. & Eskin, E. Population structure in genetic studies: confounding factors and mixed models. *PLoS Genet.* **14**, e1007309 (2018). 30589851 10.1371/journal.pgen.1007309 PMC6307707

[36] Deborah, L. GTEx project maps wide range of normal human genetic variation. *Am. J. Med. Genet. Part. A*. **176**, 263–264 (2018). 29334591 10.1002/ajmg.a.38426

[37] Zhbannikov, I. Y., Arbeev, K., Ukraintseva, S. & Yashin, A. I. haploR: an R package for querying web-based annotation tools. *F1000Research* **6**, 97 (2017). 10.12688/f1000research.10742.1 PMC5461892 28620455

[38] ENCODE Project Consortium. Expanded encyclopaedias of DNA elements in the human and mouse genomes. *Nature* **583**, 699–710 (2020). 32728249 10.1038/s41586-020-2493-4 PMC7410828

[39] Boyes, J. & Bird, A. DNA methylation inhibits transcription indirectly via a methyl-CpG binding protein. *Cell* **64**, 1123–1134 (1991). 2004419 10.1016/0092-8674(91)90267-3

[40] Newell-Price, J., Clark, A. J. & King, P. DNA methylation and silencing of gene expression. *Trends Endocrinol. Metab.* **11**, 142–148 (2000). 10754536 10.1016/s1043-2760(00)00248-4

[41] Chatterjee, R. & Vinson, C. CpG methylation recruits sequence specific transcription factors essential for tissue specific gene expression. *Biochim. Biophys. Acta*. **1819**, 763–770 (2012). 22387149 10.1016/j.bbagrm.2012.02.014 PMC3371161

[42] Wu, Y. et al. Promoter hypermethylation promotes the binding of transcription factor NFATc1, triggering oncogenic gene activation in pancreatic cancer. *Cancers (Basel)* **13**, (2021). 10.3390/cancers13184569 PMC8471171 34572796

[43] Knoll, R. et al. Identification of drug candidates targeting monocyte reprogramming in people living with HIV. *Front. Immunol.* **14**, (2023). 10.3389/fimmu.2023.1275136 PMC10703486 38077315

[44] Ssemwanga, D. et al. Multiple HIV-1 infections with evidence of recombination in heterosexual partnerships in a low risk rural clinical cohort in Uganda. *Virology* **411**, 113–131 (2011). 21239033 10.1016/j.virol.2010.12.025 PMC3041926

[45] Price, A. L. et al. Principal components analysis corrects for stratification in genome-wide association studies. *Nat. Genet.* **38**, 904–909 (2006). 16862161 10.1038/ng1847

[46] Purcell, S. et al. PLINK: a tool set for whole-genome association and population-based linkage analyses. *Am. J. Hum. Genet.* **81**, 559–575 (2007). 17701901 10.1086/519795 PMC1950838

[47] Roadmap Epigenomics Consortium. Integrative analysis of 111 reference human epigenomes. *Nature* **518**, 317–330 (2015). 25693563 10.1038/nature14248 PMC4530010

[48] Guan, Y. & Stephens, M. Practical issues in imputation-based association mapping. *PLoS Genet.* **4**, e1000279 (2008). 19057666 10.1371/journal.pgen.1000279 PMC2585794

[49] Barrett, J. C., Fry, B., Maller, J. & Daly, M. J. Haploview: analysis and visualization of LD and haplotype maps. *Bioinformatics* **21**, 263–265 (2005). 15297300 10.1093/bioinformatics/bth457

[50] Barrett, J. C. & Haploview Visualization and analysis of SNP genotype data. *Cold Spring Harb. Protoc.* **2009**, pdb.ip71 (2009). 10.1101/pdb.ip71 20147036

[51] Gabriel, S. B. et al. The structure of haplotype blocks in the human genome. *Science* **296**, 2225–2229 (2002). 12029063 10.1126/science.1069424

[52] DeGorter, M. K. et al. Transcriptomics and chromatin accessibility in multiple African population samples. *bioRxiv Prepr Serv. Biol.*10.1101/2023.11.04.564839 (2023).

[53] Mogil, L. S. et al. Genetic architecture of gene expression traits across diverse populations. *PLoS Genet.* **14**, e1007586 (2018). 30096133 10.1371/journal.pgen.1007586 PMC6105030

[54] Shang, L. et al. meQTL mapping in the GENOA study reveals genetic determinants of DNA methylation in African americans. *Nat. Commun.* **14**, 2711 (2023). 37169753 10.1038/s41467-023-37961-4 PMC10175543

[55] Boyle, A. P. et al. Annotation of functional variation in personal genomes using RegulomeDB. *Genome Res.* **22**, 1790–1797 (2012). 22955989 10.1101/gr.137323.112 PMC3431494

[56] Bundo, M. et al. A systematic evaluation of whole genome amplification of bisulfite-modified DNA. *Clin. Epigenetics*. **4**, 22 (2012). 23174095 10.1186/1868-7083-4-22 PMC3536718

[57] Gamazon, E. R. et al. A gene-based association method for mapping traits using reference transcriptome data. *Nat. Genet.* **47**, 1091–1098 (2015). 26258848 10.1038/ng.3367 PMC4552594

