# Supplementary material for: Functionally-informed fine-mapping identifies genetic variants linking increased CHD1L expression and HIV restriction in monocytes
Source: Sci Rep. 2025 Jan 17;15:2325. doi: 10.1038/s41598-024-84817-y (PMC11748618; doi:10.1038/s41598-024-84817-y)
Supplement: Supplementary file 2 — Supplementary Material 2 [file 41598_2024_84817_MOESM2_ESM.pdf]

Supplementary Note: Cohorts and individuals contributing to the International Consortium for the Genomics of HIV (ICGH)

The AIDS Clinical Trials Group (ACTG)

DESCRIPTION:

The AIDS Clinical Trials Group (ACTG) was initially established in 1987 to broaden the scope of the AIDS research effort of the US National Institute of Allergy and Infectious Diseases (NIAID). The ACTG established and supports the largest Network of expert clinical and translational investigators and therapeutic clinical trials units in the world, including sites in resource-limited countries. These investigators and units serve as the major resource for HIV/AIDS research, treatment, care, and training/education in their communities.

MEMBERS:

Eric S. Daar, Roy M. Gulick, David W. Haas, Richard Haubrich, Daniel R. Kuritzkes, Heather J. Ribaudo, Sharon Riddler, Gregory K. Robbins, Paul E. Sax, Robert W. Shafer, Cecilia M. Shikuma

FUNDING:

Research reported in this publication was supported by the National Institute of Allergy and Infectious Diseases of the National Institutes of Health under Award Number UM1 AI068634, UM1 AI068636 and UM1 AI106701. The content is solely the responsibility of the authors and does not necessarily represent the official views of the National Institutes of Health. Grants to Clinical Research Sites that participated in ACTG protocols and collected DNA under protocol A5128 included: AI069477, AI027675, AI073961, AI069474, AI069432, AI069513, AI069423, AI050410, AI069452, AI69450, AI054907, AI069428, AI045008, AI069495, AI069415, AI069556, AI069484, AI069424, AI069532, AI069419, AI069471, AI025859, AI069418,

AI050409, AI069501, AI069502, AI069511, AI069434, AI069465, AI069494, AI069472, AI069470, AI046376, AI072626, AI027661, AI034853, AI069447, AI032782, AI027658, AI27666, AI058740, AI046370, TR002243, RR00051, RR00046, RR025747, RR025777, RR024160, RR024996, and RR024156. Abbott Laboratories, Boehringer-Ingelheim, Bristol-Myers Squibb, Gilead Sciences, and GlaxoSmithKline provided study medications. DWH is also supported by NIH grants AI077505 and AI110527.

ETHICS:

This study was approved by the Institutional Review Board at Vanderbilt University, Nashville USA.

The AIDS Linked to the IntraVenous Experience (ALIVE) Cohort

DESCRIPTION:

The AIDS Linked to the IntraVenous Experience (ALIVE) study is a natural history study following the incidence and progression of HIV infection among intravenous drug users (IDUs) in Baltimore, MD.

MEMBERS:

Gregory D. Kirk, Shruti H. Mehta, Kenrad Nelson, Steffanie Strathdee, David Vlahov

FUNDING:

National Institutes of Health grants: R01-DA-04334 and R01-DA-12568

ETHICS:

This study was approved by the Institutional Review Board of the Johns Hopkins School of Medicine, Baltimore USA.

The Center for Cancer Research, National Cancer Institute

DESCRIPTION:

The Center for Cancer Research (CCR) is home to more than 250 scientists and clinicians working in intramural research at NCI. CCR's investigators are basic, clinical, and translational scientists who work together to advance knowledge of cancer and AIDS and to develop new therapies against these diseases.

MEMBERS:

Ping An, Sher Hendrickson, Randall Johnson, Bailey Kessing, James Lautenberger, Carl McIntosh, George Nelson, Stephen O'Brien, Efe Szegin, Jennifer Troyer, and Cheryl Winkler

FUNDING:

This research was supported by the Intramural Research Program of NIH, Frederick National Laboratory, Center for Cancer Research, National Cancer Institute and also funded in whole or in part with Federal funds from the Frederick National Laboratory for Cancer Research, National Institutes of Health, under contract HHSN261200800001E.

ETHICS:

Genetic studies of this cohort has been granted by the NIH Office of Human Subjects Research.

The Center for HIV/AIDS Vaccine Immunology (CHAVI)

DESCRIPTION:

The Center for HIV/AIDS Vaccine Immunology (CHAVI) is a consortium of universities and academic medical centers that was established by the National Institute of Allergy and Infectious Diseases (NIAID) from 2005-2012. CHAVI's goal was to solve the major problems in HIV vaccine development and design.

MEMBERS:

CHAVI is led by Barton Haynes (Duke University, Durham, NC, USA). Its Host Genetics Core is led by David Goldstein (Duke University, Durham, NC, USA).

FUNDING:

Funding for research by CHAVI was provided by NIH NIAID grant AI067854.

ETHICS:

All participating centers provided local institutional review board approval.

The Hemophilia Growth and Development Study (HGDS)

DESCRIPTION:

The HGDS is a multicenter study of hemophilia and its complications that was established in 1988. Data were prospectively collected in 14 U.S. hemophilia treatment centers through 1996/97. A total of 333 children and adolescents were enrolled. The HGDS has investigated the effects of hemophilia and HIV on physical growth and maturation; immunological, neurological, and neuropsychological functioning; and the pathophysiology of HIV and hepatitis C.

MEMBERS:

Eric Daar, Sharyne Donfield, Edward Gomperts, Margaret Hilgartner, W. Keith Hoots, Henry Lynn, Anne Willoughby, Cheryl Winkler.

FUNDING:

National Institutes of Health, National Institute of Child Health and Human Development, R01-HD-41224

ETHICS:

Genetic studies of this cohorts has been granted by the NIH Office of Human Subjects Research.

The International HIV Controllers Study

DESCRIPTION:

The International HIV Controllers Study is a collaborative effort among scientists, healthcare professionals and the community to study HIV infected people who have been able to maintain low viral loads without the use of medications.

MEMBERS:

HIV controllers recruitment and sample management: Florencia Pereyra, Alicja Piechocka-Trocha, Emily Cutrell, Rachel Rosenberg, Kristin L. Moss, Ildiko Toth, Brian Block, Brett Baker, Alissa Rothchild, Jeffrey Lian, Jacqueline Proudfoot, Marylyn M. Addo, Bruce D. Walker. HIV controllers referral team: Brian Agan, Shanu Agarwal, Richard L. Ahern, Brady L. Allen, Sherly Altidor, Eric L. Altschuler, Sujata Ambardar, Kathryn Anastos, Val Anderson, Ushan Andrady, Diana Antoniskis, David Bangsberg, Daniel Barbaro, William Barrie, J. Bartczak, Simon Barton, Patricia Basden, Nesli Basgoz, Nicholaos C. Bellos, Judith Berger, Nicole F. Bernard, Annette M. Bernard, Stanley J. Bodner, Robert K. Bolan, Emilie T.

Boudreaux, James F. Braun, Jon E. Brndjar, J. Brown, Sheldon T. Brown, Jedidiah Burack, Larry M. Bush, Virginia Cafaro, John Campbell, Robert H. Carlson, J. Kevin Carmichael, Kathleen K. Casey, Chris Cavacuiti, Gregory Celestin, Steven T. Chambers, Nancy Chez, Lisa M. Chirch, Paul J. Cimoch, Daniel Cohen, Lillian E. Cohn, Brian Conway, David A. Cooper, Brian Cornelson, David T. Cox, Michael V. Cristofano, George Cuchural Jr., Julie L. Czartoski, Joseph M. Dahman, Jennifer S. Daly, Benjamin T. Davis, Kristine Davis, Sheila M. Davod, Steven G. Deeks, Edwin DeJesus, Craig A. Dietz, Eleanor Dunham, Michael E. Dunn, Todd B. Ellerin, Joseph J. Eron, John J.W. Fangman, Helen Ferlazzo, Sarah Fidler, Anita Fleenor-Ford, Renee Frankel, Kenneth A. Freedberg, Neel K. French, Jonathan D. Fuchs, Jon D. Fuller, Jonna Gaberman, Joel E. Gallant, Rajesh T. Gandhi, Efrain Garcia, Donald Garmon, Joseph C. Gathe Jr, Cyril R. Gaultier, Wondwoosen Gebre, Frank D. Gilman, Ian Gilson, Paul A. Goepfert, Michael S. Gottlieb, Claudia Goulston, Richard K. Groger, T. Douglas Gurley, Stuart Haber, Robin Hardwicke, W. David Hardy, P. Richard Harrigan, Trevor N. Hawkins, Sonya Heath, Frederick M. Hecht, W. Keith Henry, Melissa Hladek, Robert P. Hoffman, James M. Horton, Ricky K. Hsu, Gregory D. Huhn, Peter Hunt, Mark L. Illeman, Hans Jaeger, Robert M. Jellinger, Mina John, Jennifer A. Johnson, Kristin L. Johnson, Heather Johnson, Kay Johnson, Jennifer Joly, Wilbert C. Jordan, Carol A. Kauffman, Homayoon Khanlou, Arthur Y. Kim, David D. Kim, Clifford A. Kinder, Laura Kogelman, Erna Milunka Kojic, Neeltje A. Kootstra, P. Todd Korthuis, Wayne Kurisu, Douglas S. Kwon, Melissa LaMar, Harry Lampiris, Michael M. Lederman, David M. Lee, Marah J. Lee, Edward T.Y. Lee, Janice Lemoine, Jay A. Levy, Josep M. Llibre, Michael A. Liguori, Susan J. Little, Anne Y. Liu, Alvaro J. Lopez, Mono R. Loutfy, Dawn Loy, Debbie Y. Mohammed, Alan Man, Michael K. Mansour, Vincent C. Marconi, Martin Markowitz, Jeffrey N. Martin, Harold L. Martin Jr., Kenneth Hugh Mayer, M. Juliana McElrath,

Theresa A. McGhee, Barbara H. McGovern, Katherine McGowan, Dawn McIntyre, Gavin X. McLeod, Prema Menezes, Greg Mesa, Craig E. Metroka, Dirk Meyer-Olson, Andy O. Miller, Kate Montgomery, Karam C. Mounzer, Iris Nagin, Ronald G. Nahass, Craig Nielsen, David L. Norene, David H. O'Connor, Jason Okulicz, Edward C. Oldfield III, Susan A. Olender, Mario Ostrowski, William F. Owen Jr., Jeffrey Parsonnet, Andrew M. Pavlatos, Aaron M. Perlmutter, Jonathan M. Pincus, Leandro Pisani, Lawrence Jay Price, Laurie Proia, Richard C. Prokesch, Heather Calderon Pujet, Moti Ramgopal, Michael Rausch, J. Ravishankar, Frank S. Rhame, Constance Shamuyarira Richards, Douglas D. Richman, Gregory K. Robbins, Berta Rodes, Milagros Rodriguez, Richard C. Rose III, Eric S. Rosenberg, Daniel Rosenthal, Polly E. Ross, David S. Rubin, Elease Rumbaugh, Luis Saenz, Michelle R. Salvaggio, William C. Sanchez, Veeraf M. Sanjana, Steven Santiago, Wolfgang Schmidt, Philip M. Sestak, Peter Shalit, William Shay, Vivian N. Shirvani, Vanessa I. Silebi, James M. Sizemore Jr., Paul R. Skolnik, Marcia Sokol-Anderson, James M. Sosman, Paul Stabile, Jack T. Stapleton, Francine Stein, Hans-Jurgen Stellbrink, F. Lisa Sterman, Valerie E. Stone, David R. Stone, Giuseppe Tambussi, Randy A. Taplitz, Ellen M. Tedaldi, Amalio Telenti, Richard Torres, Lorraine Tosiello, Cecile Tremblay, Marc A. Tribble, Phuong D. Trinh, Anthony Vaccaro, Emilia Valadas, Thanes J. Vanig, Isabel Vecino, Wenoah Veikley, Barbara H. Wade, Charles Walworth, Chingchai Wanidworanun, Douglas J. Ward, Robert D. Weber, Duncan Webster, Steve Weis, David A. Wheeler, David J. White, Ed Wilkins, Alan Winston, Clifford G. Wlodaver, David P. Wright, Otto O. Yang, David L. Yurdin, Brandon W. Zabukovic, Kimon C. Zachary, Beth Zeeman, Meng Zhao

FUNDING:

The International HIV Controllers Study was made possible through a generous donation from the Mark and Lisa Schwartz Foundation and a subsequent award from the Collaboration for

AIDS Vaccine Discovery (CAVD) of the Bill and Melinda Gates Foundation. This work was also supported in part by the Harvard University Center for AIDS Research (P-30- AI060354), UCSF CFAR (P-30 AI27763), UCSF CTSI (UL1 RR024131), CNICS (R24 AI067039), NIH grants AI28568, AI030914 (B.D.W.); AI087145, K24AI069994 (S.G.D.)

ETHICS:

HIV controllers were recruited through local outpatient clinics affiliated with the Ragon Institute of MGH, MIT and Harvard and collaborations with 335 health care providers and scientists across the US, Canada, Western Europe and Australia. The respective institutional review boards approved the study, and all subjects gave written informed consent.

The Multicenter AIDS Cohort Study (MACS)

DESCRIPTION:

The Multicenter AIDS Cohort Study (MACS) is an ongoing prospective study of the natural and treated histories of HIV-1 infection in homosexual and bisexual men conducted by sites located in Baltimore, Chicago, Pittsburgh and Los Angeles. A total of 6,972 men have been enrolled.

MEMBERS:

Baltimore: The Johns Hopkins University Bloomberg School of Public Health: Joseph B. Margolick (Principal Investigator), Barbara Crain, Adrian Dobs, Homayoon Farzadegan, Joel Gallant, Lisette Johnson, Shenghan Lai, Ned Sacktor, Ola Selnes, James Shepherd, Chloe Thio.

Chicago: Howard Brown Health Center, Feinberg School of Medicine, Northwestern University, and Cook County Bureau of Health Services: John P. Phair and Steven Wolinsky (Multiple

Principal Investigators), Sheila Badri, Bruce Cohen, Craig Conover, Maurice O'Gorman, David Ostrow, Frank Palella.

Los Angeles: University of California, UCLA Schools of Public Health and Medicine: Roger Detels (Principal Investigator), Barbara R. Visscher (Co-Principal Investigator), Aaron Aronow, Robert Bolan, Elizabeth Breen, Anthony Butch, Thomas Coates, Rita Effros, John Fahey, Beth Jamieson, Otoniel Martínez-Maza, Eric N. Miller, John Oishi, Paul Satz, Harry Vinters, Dorothy Wiley, Mallory Witt, Otto Yang, Stephen Young, Zuo Feng Zhang.

Pittsburgh: University of Pittsburgh, Graduate School of Public Health: Charles R. Rinaldo (Principal Investigator), Lawrence Kingsley (Co-Principal Investigator), James T. Becker, Robert W. Evans, John Mellors, Sharon Riddler, Anthony Silvestre.

Data Coordinating Center: The Johns Hopkins University Bloomberg School of Public Health: Lisa P. Jacobson (Principal Investigator), Alvaro Muñoz (Co-Principal Investigator), Keri Althoff, Christopher Cox, Gypsyamber D'Souza, Stephen J. Gange, Elizabeth Golub, Janet Schollenberger, Eric C. Seaberg, Sol Su.

FUNDING:

NIH: National Institute of Allergy and Infectious Diseases: Robin E. Huebner; National Cancer Institute: Geraldina Dominguez; National Heart, Lung and Blood Institute: Cheryl McDonald. UO1-AI-35042, 5-M01-RR-00052 (GCRC), UO1-AI-35043, UO1-AI37984, UO1-AI-35039, UO1-AI-35040, UO1-AI-37613, and UO1-AI-35041. This work was also supported in part by NIH grants R37 AI47734 (J.I.M.); T32 AI07140 (J.T.H.), and the University of Washington Center for AIDS Research, an NIH funded program (P30 AI027757), which is supported by the following NIH Institutes and Centers (NIAID, NCI, NIMH, NIDA, NICHD, NHLBI, NIA).

ETHICS:

This study was approved by the Institutional Review Boards at Northwestern University, Chicago USA; University of California at Los Angeles, Los Angele USA; University of Pittsburgh, Pittsburgh USA; and Johns Hopkins University, Baltimore USA.

The Multicenter Hemophilia Cohort Studies (MHCS)

DESCRIPTION:

The first Multicenter Hemophilia Cohort Study (MHCS-I) evaluated and prospectively followed patients with hemophilia or a related coagulation disorder. Initiated in 1982, this study particularly sought to understand the cause and natural history of HIV infection and AIDS in this population, which was at high risk for development of AIDS.

MEMBER:

James J. Goedert.

FUNDING:

Intramural Research Program, National Cancer Institute, National Institutes of Health

ETHICS:

Genetic studies of this cohorts has been granted by the NIH Office of Human Subjects Research.

The Pumwani Sex Workers Cohort

DESCRIPTION:

The Nairobi (Pumwani) commercial sex worker cohort was established in 1985. Despite repeated exposures to HIV-1, a number of women in this cohort have remained HIV-1 uninfected for long periods of time and have been epidemiologically defined as HIV resistant.

MEMBERS:

Francis A. Plummer, Terry Blake Ball, Keith Fowke, Joshua Kimani, Larry Gelmon, Ma Luo, Elizabeth Ngugi

FUNDING:

The Pumwani Sex Workers Cohort has been supported by various grants from Canadian Institute of Health Research, Canadian International Development Agency, National Institute of Health (R01 AI56980), USA and Bill and Melinda Gates Foundation since 1985. This work was supported by a grant from the Bill and Melinda Gates Foundation and the Canadian Institutes of Health Research (HOP-43135) through the Grand Challenges in Global Health Initiative.

ETHICS:

This study was approved by the Institutional Review Board of the University of Manitoba, Winnipeg Canada and Kenyatta National Hospital, Nairobi, Kenya.

The San Francisco City Clinic Cohort (SFCCC)

DESCRIPTION:

The San Francisco City Clinic Cohort (SFCCC) consists of approximately 6700 homosexual men recruited between 1978 and 1980 from a clinic for sexually transmitted diseases.

MEMBERS:

Susan Buchbinder

FUNDING:

This was supported by cooperative agreement (No U62/CCU900523) from the Centers for Disease Control, Atlanta, Georgia

ETHICS:

This study was approved by the Institutional Review Board of the State of California Health and Human Services Agency Committee for the Protection of Human Subjects

The Swiss HIV Cohort Study (SHCS)

DESCRIPTION:

The Swiss HIV Cohort Study (SHCS) is an ongoing multi-center research project dealing with HIV infected adults aged 16 years or older. Since it was established in 1988, the SHCS has recruited and followed more than 17,000 patients in seven centers. The data are gathered by the Five Swiss University Hospitals, two Cantonal Hospitals, 15 affiliated hospitals and 36 private physicians (listed in http://www.shcs.ch/180-health-care-providers).

MEMBERS:

Aebi-Popp K, Anagnostopoulos A, Battegay M, Bernasconi E, Böni J, Braun DL, Bucher HC, Calmy A, Cavassini M, Ciuffi A, Dollenmaier G, Egger M, Elzi L, Fehr J, Fellay J, Furrer H, Fux CA, Günthard HF (President of the SHCS), Haerry D (deputy of "Positive Council"), Hasse B, Hirsch HH, Hoffmann M, Hösli I, Huber M, Kahlert CR (Chairman of the Mother & Child Substudy), Kaiser L, Keiser O, Klimkait T, Kouyos RD, Kovari H, Ledergerber B, Martinetti G, Martinez de Tejada B, Marzolini C, Metzner KJ, Müller N, Nicca D, Paioni P, Pantaleo G,

Perreau M, Rauch A (Chairman of the Scientific Board), Rudin C, Scherrer AU (Head of Data Centre), Schmid P, Speck R, Stöckle M (Chairman of the Clinical and Laboratory Committee), Tarr P, Trkola A, Vernazza P, Wandeler G, Weber R, Yerly S.

FUNDING:

This study has been financed within the framework of the Swiss HIV Cohort Study, supported by the Swiss National Science Foundation (grant #201369), by SHCS project #841 and by the SHCS research foundation. The data are gathered by the Five Swiss University Hospitals, two Cantonal Hospitals, 15 affiliated hospitals and 36 private physicians (listed in http://www.shcs.ch/180-health-care-providers).

ETHICS:

This study was approved by the Institutional Review Board of the participating centres: Ethikkommission beider Basel; Kantonale Ethikkommission Bern; Comité départemental d'éthique des spécialités médicales et de médecine communautaire et de premier recours, Hôpitaux Universitaires de Genève; Commission cantonale d'éthique de la recherche sur l'être humain, Canton de Vaud; Comitato etico cantonale, Repubblica e Cantone Ticino; Ethikkommission des Kantons St. Gallen; Kantonale Ethikkommission Zürich

Urban Health Study: Genetics Cohort (UHSGC)

DESCRIPTION:

The Urban Health Study was a serial, cross-sectional sero-epidemiological study. Data were collected every 6 months in communities with a high prevalence of injection drug use: 35 semi-annual “waves” across 19 years of data collection in the San Francisco Bay Area CA U.S. (1986-

2005). Eligibility criteria included having injected drugs in the past 30 days, as confirmed by visual inspection. From the UHS we have banked biospecimens from about 15,000 people who injected drugs (PWIDs). From these UHS participants we developed the UHSGC, genotyping selected HIV+ cases and exposure-matched HIV- controls (total N = 3,136).

MEMBERS:

Laura J. Bierut, Nathan C. Gaddis, Cristie Glasheen, Dana B. Hancock, Eric O. Johnson (PI: UHSGC), Alex H. Kral (PI: UHS), Joshua L. Levy, Grier Page, Nancy L. Saccone

FUNDING:

U.S. National Institute on Drug Abuse (grants R01DA026141, R01DA038632).

ETHICS:

This study was approved by the Research Triangle Institute (RTI) Institutional Review Board under the RTI International Office of Research Protection, North Carolina USA.

The US military HIV Natural History Study (NHS)

DESCRIPTION:

The US military HIV Natural History Study is a longitudinal epidemiological, observational, open cohort study collecting retrospective and prospective data in the U.S. Military active duty and Department of Defense (DoD) health care beneficiary HIV infected population.

MEMBERS:

Brian Agan, Mary Bavaro, Helen Chun, Nancy Crum-Cianflone, Cathy Decker, Connor Eggleston, Tomas Ferguson, Susan Fraser, Anuradha Ganesan, Joshua Hartzell, Joshua Hawley,

Gunther Hsue, Arthur Johnson, Mark Kortepeter, Tahaniyat Lalani, Grace Macalino, Scott Merritt, Robert O'Connell, Jason Okulicz, Sheila Peel, Michael Polis, John Powers, Rose Ressner, Edmund Tramont, Tyler Warkentien, Amy Weintrob, Timothy Whitman, Michael Zapor

ETHICS:

This study was approved by the Institutional Review Board of the Uniformed Services University of the Health Sciences, Bethesda USA

FUNDING:

Support for this work was provided by the Infectious Disease Clinical Research Program (IDCRP), a Department of Defense (DoD) program executed through the Uniformed Services University of the Health Sciences. This project has been funded in whole, or in part, with federal funds from the National Institute of Allergy and Infectious Diseases, National Institutes of Health (NIH), under Inter-Agency Agreement Y1-AI-5072. The content of this publication is the sole responsibility of the authors and does not necessarily reflect the views or policies of the NIH or the Department of Health and Human Services, the DoD or the Departments of the Army, Navy or Air Force. Mention of trade names, commercial products, or organizations does not imply endorsement by the U.S. Government.

The Rural clinical cohort (RCC)

DESCRIPTION:

The Rural clinical cohort (RCC) is an open clinical cohort established in 1990 by the Medical Research Council (MRC), UK in collaboration with the Uganda Virus Research Institute

(UVRI), to study the natural history of HIV infection and later the impact of ART after its introduction in 2004. Full details of the cohort structure and the annual HIV survey have been previously published.

MEMBERS:

Anatoli Kamali (formerly UVRI), Pontiano Kaleebu (UVRI), Manjinder Sandhu (WSI)

FUNDING:

Support for this work was provide by the UK Medical Research Council (grants G0901213-92157, G0801566, and MR/K013491/1), core funding of the collaboration between MRC and UVRI as well as Wellcome (WT098051)

ETHICS:

This study was approved by the Uganda Virus Research Institute Science Ethics Committee and the IRB of the Uganda National Council for Science and Technology.

International AIDS Vaccine Initiative (IAVI)

DESCRIPTION:

The International AIDS Vaccine Initiative (IAVI) is the nonprofit organization dedicated to accelerating development of vaccines to prevent AIDS.

MEMBERS:

Matthew A Price (IAVI/UCSF)

FUNDING:

This work was funded in part by IAVI with the generous support of the United States Agency for International Development (USAID) and other donors. The full list of IAVI donors is available at http://www.iavi.org. The contents of this manuscript are the responsibility of IAVI and co-authors and do not necessarily reflect the views of USAID or the US Government.

ETHICS:

This study was approved by the following Institutional Review Boards: the Kenya Medical Research Institute Ethical Review Committee, the Kenyatta National Hospital Ethical Review Committee of the University of Nairobi, the Rwanda National Ethics Committee, the Uganda Virus Research Institute Science and Ethics Committee, the Uganda National Council of Science and Technology, the University of Cape Town Health Science Research and Ethics Committee, the University of Zambia Research Ethics Committee, the Bio-Medical Research Ethics Committee at the University of KwaZulu Natal, and the Emory University Institutional Review Board.

The African Transcriptome Resource (ATR)

DESCRIPTION:

The African Transcriptome Resource (ATR) has been established to develop a transcriptomics resource across diverse populations in Africa and to facilitate a better understanding of the genetic diversity and functional consequences on gene expression across African populations. To date this resource includes data generated from 1000 Genomes Project populations.

MEMBERS:

Manjinder Sandhu (Wellcome Sanger Institute), Paul Flicek (European Bioinformatics Institute), Jacques Fellay (École Polytechnique Fédérale de Lausanne), Paul McLaren (University of Manitoba), Stephen Montgomery (Stanford University)

FUNDING:

This work was funded by Wellcome (WT206194), IAVI's USAID Cooperative Agreement (AID-OAA-A-16-00032), the European Molecular Biology Laboratory, the École Polytechnique Fédérale de Lausanne, the Public Health Agency of Canada and Stanford University.
